# Response to Sachdeva et al: Brain Fogginess and SIBO Is Not a Mirage

**DOI:** 10.1038/s41424-018-0061-0

**Published:** 2018-10-05

**Authors:** Satish S. C. Rao, Siegfried Yu

**Affiliations:** Division of Gastroenterology/Hepatology, Department of Internal Medicine, Medical College of Georgia, Augusta University, Augusta, GA USA

We thank Prof. Sachdeva and colleagues for their useful comments, and pleased that they found this article “fascinating” and “thought provoking”, and happy to respond to their concerns. (1) Is Brain Fogginess a center-point to SIBO? We believe that BF is a feature in a select group of SIBO patients in whom the small bowel is colonized with d-lactic acid producing organisms, particularly lactobacillus species, contained in probiotics. We emphasize that not all SIBO patients have BF or colonization with these organisms. Also, with only seven publications on BF, Sachdeva et al. have accepted that BF occurs in POTS, fibromyalgia, etc.; but are suspicious of this in SIBO; it is time that gastroenterologists recognize that BF can be a manifestation of SIBO. (2) Objective test for BF? There is no definition or validated questionnaire or objective test for BF according to neurologists we have discussed, and hence could not perform any tests. We are currently validating a questionnaire. (3) Is BF and SIBO linked? Absolutely, and was the essence of our study. BF was reproduced in 66% of subjects during the breath test, and most of them had evidence of SIBO (GBT or culture). The lower yield (sensitivity) of GBT and duodenal aspirate is consistent with multiple other studies^1,2,3^, as they only measure proximal SIBO and miss distal SIBO. (4) BF not related to lactic acidosis? Our patients did not report BF 24 h a day. It would wax and wane, but a majority experienced this postprandially. Hence, we tested them with a GBT; both to assess whether BF can be reproduced in the lab, and to validate that it was not an imaginary or supratentorial (functional) symptom, as well as to identify its association with SIBO. The fact that in almost all in whom the urinary d-Lactic acid increased did occur after a glucose meal and was associated with BF proves the link between BF and d-lactic acidosis. (5) Lack of reproducibility of BF on breath testing challenges link with SIBO? As discussed above and by Rezai et al. in North American Consensus^3^, current modalities for SIBO testing are imperfect. The small bowel is 19 ft long, and can be colonized anywhere. In contrast, glucose when administered orally is absorbed within the first 3–6 ft of small bowel, and duodenal aspirates are typically from the proximal 2 ft of small bowel. Hence, to rely on glucose breath testing alone to make a diagnosis of SIBO will provide a low yield of about 33% and miss many other patients. This was a reason why we used two tests, both GBT and duodenal aspirates to maximize the detection of SIBO in our patients. Therefore, the author’s contention that many patients with a negative GBT had D-lactic acidosis and/or BF makes it unlikely that they had SIBO, is inaccurate.

Finally, we would encourage them to use our protocol and identify this syndrome in their patient population, and relieve their suffering, and yes we are now performing a post-treatment study.

## References

[1] Jacobs C, Coss-Adame E, Attaluri A, Valestin J, Rao S.S. Dysmotility and proton pump inhibitor use are independent risk factors for small intestinal bacterial and/or fungal overgrowth. *Aliment. Pharmacol. Ther*. **37,** 1103–1111 (2013). 10.1111/apt.12304 PMC3764612 23574267

[2] Erdogan A. et al. Small Intestinal Bacterial Overgrowth: Duodenal aspiration vs. glucose breath test. *Neurogastroenterol Motil*. **27**, 481–489 (2015). 10.1111/nmo.12516 25600077

[3] Rezaie A. et al. Hydrogen and methane-based breath testing in gastrointestinal disorders: The North American Consensus. *Am J Gastroenterol*. **112**, 775–785 (2017). 10.1038/ajg.2017.46 PMC5418558 28323273

